# Population genetic structure of a Chihuahuan Desert endemic mammal, the desert pocket gopher, *Geomys arenarius*

**DOI:** 10.1002/ece3.10576

**Published:** 2023-09-28

**Authors:** Russell S. Pfau, Ashley N. Kozora, Ana B. Gatica‐Colima, Philip S. Sudman

**Affiliations:** ^1^ Department of Biological Sciences Tarleton State University Stephenville Texas USA; ^2^ Cooper High School Abilene Texas USA; ^3^ Departamento de Ciencias Químico‐Biológicas Instituto de Ciencias Biomédicas, Universidad Autónoma de Ciudad Juárez Ciudad Juarez Mexico

**Keywords:** AFLP, conservation genetics, mtDNA, phylogeography, population genetics, soil

## Abstract

The biogeographic history of the Chihuahuan Desert is complex, driven by numerous physiographic events and climatic changes. This dynamic history would have influenced the flora and fauna of the region including the desert pocket gopher, *Geomys arenarius*, a subterranean rodent endemic to the northern Chihuahuan Desert. *G*. *arenarius* is restricted to sandy soils and are considered to have a disjunct distribution. Two subspecies are recognized: *G*. *a*. *arenarius* and *G*. *a*. *brevirostris*. We used multilocus nuclear (amplified fragment length polymorphisms) and mitochondrial DNA (ND2) sequence data to uncover patterns of genetic diversity within and among populations of *G*. *arenarius*. We evaluated correspondence of genetic patterns to traditionally accepted subspecies boundaries, mapped the distribution of potentially suitable soils to identify barriers or corridors to dispersal and to guide future survey efforts, provided evidence that could be used to recognize distinct population segments, and quantified genetic diversity within populations. Both datasets were largely concordant and demonstrated hierarchical patterns of genetic divergence. The greatest divergence was consistent with the two recognized subspecies. Mapping of potentially habitable soils revealed likely barriers to dispersal contributing to the allopatric pattern of geographic distribution and areas, which may be occupied by *G*. *arenarius* but not yet documented. Because *G*. *arenarius* is restricted to soils with high sand content, and these habitable soils are disjunct within the region occupied by this species, historical factors that impacted soil deposition and deflation likely contributed to the observed patterns of genetic divergence. Genetic diversity was higher within populations of the southern subspecies (*G*. *a*. *arenarius*) compared to *G*. *a*. *brevirostris*. This may be due to a greater availability of continuous suitable soils within the range of *G*. *a*. *arenarius* or higher density due to greater food availability (currently or historically)—both of which could allow for a higher effective population size.

## Graphical abstract

The desert pocket gopher, *Geomys arenarius*, is a burrowing mammal endemic to the biogeographically complex Chihuahuan Desert. Multilocus nuclear and mitochondrial DNA sequence data revealed concordant population structure within this species. Distribution of likely habitable soils is consistent with population structure, and the population structure is consistent with current subspecific taxonomy.

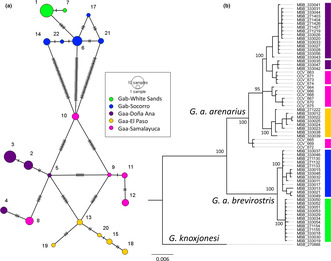

## 1 INTRODUCTION

The Chihuahuan Desert of the southwestern United States and northern Mexico is geologically diverse, consisting of mountain ranges, valleys and basins with gravelly and sandy soils, areas of extensive sand dunes, and playas within closed basins. Considered to be among the world's most biologically diverse deserts (Ricketts et al., 1999), this unique landscape has experienced many changes over recorded and historical times. Grasslands have largely been replaced by shrublands over the past 150 years (Gibbens et al., 2005; Hennessy et al., 1983; Yanoff & Muldavin, 2008; York & Dick‐Peddie, 1969) and many areas along the Rio Grande have been modified for agricultural and urban use. In addition to recent vegetational changes, the Chihuahuan Desert region saw intense geomorphological and climatic changes during the Neogene and Pleistocene periods, but the timing of these events and their effect on the biota are uncertain (Wilson & Pitts, 2010). These changes are considered to have impacted the distribution and genetic structure of several vertebrate species (Castellanos‐Morales et al., 2016; Díaz‐Cárdenas et al., 2019; Hafner & Riddle, 2005; Jaeger et al., 2005; Neiswenter & Riddle, 2010; Riddle, 1995; Riddle et al., 2000). Despite the high biological and geological diversity of the Chihuahuan Desert, relatively few studies have examined the population genetic structure of species endemic to this region.

Subterranean mammals, because their occurrence is limited to suitable soils, are likely to have been impacted by geomorphological and climatic changes in ways that reflect processes, which affected their dispersal across the landscape. Four species of subterranean mammals within the family Geomyidae occupy the Chihuahuan Desert: *Thomomys bottae*, *T*. *umbrinus*, *Cratogeomys castanops*, and *Geomys arenarius*. Of these four, only *G*. *arenarius* is endemic to the Chihuahuan Desert, ranging from south‐central New Mexico southward into western‐most Texas and the northern part of the Mexican state of Chihuahua (Williams & Baker, 1974). *G*. *arenarius* has only been collected in relatively sandy soils and its distribution consists of multiple, apparently disjunct populations (Davis, 1940; Fernández et al., 2014; Hafner & Geluso, 1983). Their anatomy, adapted for shearing and pushing soil, renders aboveground locomotion clumsy and inefficient and likely increases risk of predation. Vleck (1979) demonstrated that, in gophers of the genus *Thomomys*, the energetic cost of burrowing was much higher than surface locomotion and that the cost of burrowing in clay soil was considerably higher than in sandy soils. Pocket gophers (*Geomyidae*) feed mostly from their underground tunnels and are generalist herbivores. *Geomys arenarius* in Mexico was documented to consume both monocots and dicots in 10 plant families (Rueda‐Torres et al., 2022). *G*. *attwateri*, a species closely related to *G*. *arenarius*, consumed 36 of the 51 plant species available to them (Williams & Cameron, 1986). Another closely related species (*G*. *bursarius*) consumed mostly grasses (of several genera) along with the cactus *Opuntia* (Luce et al., 1980; Myers & Vaughan, 1965).

**FIGURE 1:**
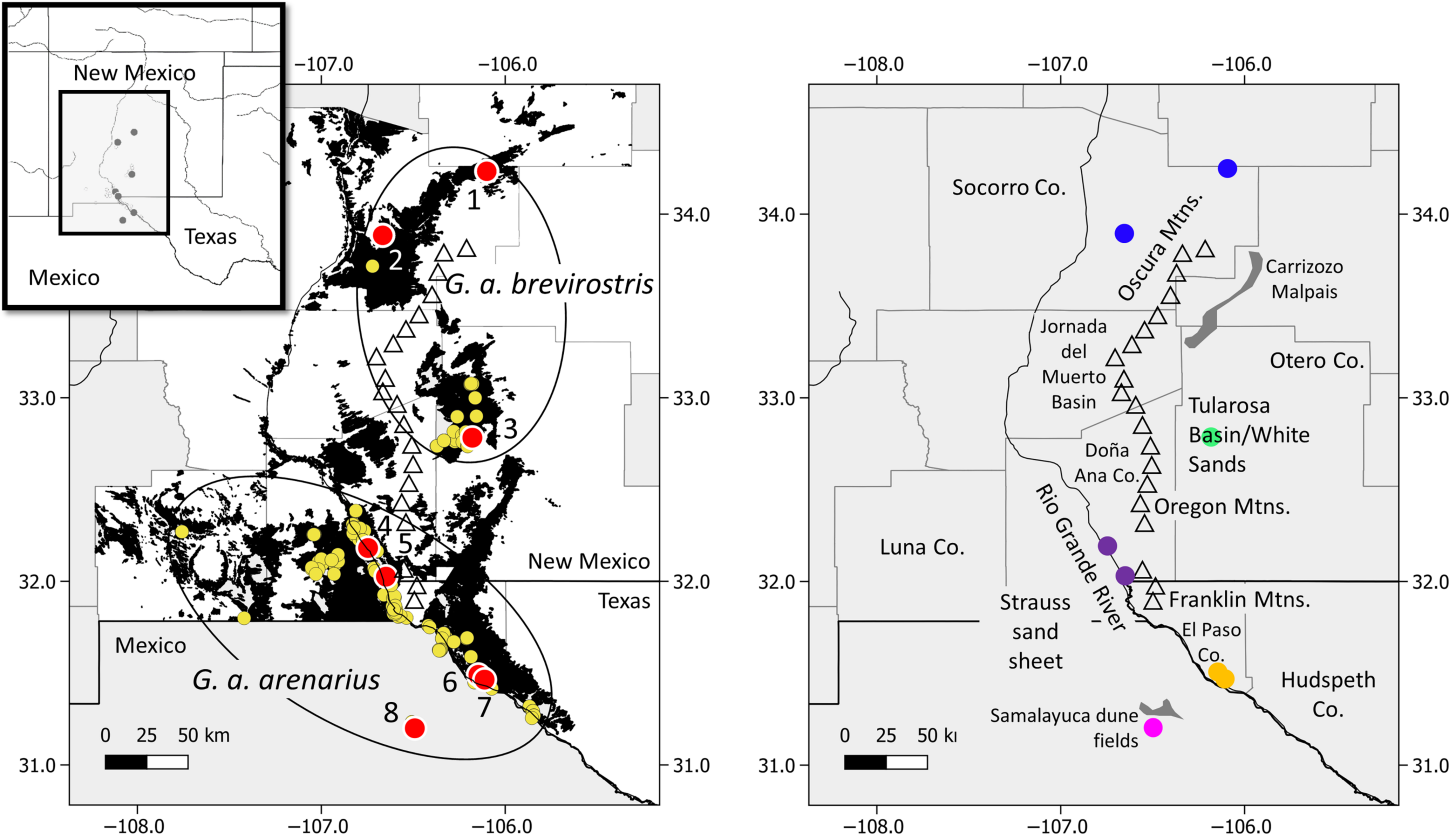
Documented occurrence of *Geomys arenarius* based on voucher specimens in museum databases (yellow symbols), collection localities for this study (red symbols), and geographic features mentioned within the text. Numbers for collection localities correspond to those in Appendix S1: 1—Gran Quivira, Socorro Co., NM; 2—Near San Antonio, Socorro Co., NM; 3—White Sands National Monument, Otero Co., NM; 4 and 5— Doña Ana Co., NM; 6 and 7—El Paso Co., TX; 8—Samalayuca, MX. Ovals group locations by currently recognized subspecies (sensu Hafner & Geluso, 1983). Predicted areas of suitable soils are shown in black (only counties shown in white were included in the soil analysis). Triangles indicate mountain ranges within the geographic distribution of the species. Locality symbols on the right‐hand map are color coded to match Figures 2 and 5.

Hall (1932) recognized two subspecies, *G*. *a*. *arenarius* and *G*. *a*. *brevirostris* based on morphological differences between specimens from the vicinity of White Sands National Monument, NM (Otero Co.; hereafter referred to as White Sands) compared to those from further south. Later, Williams and Genoways (1978) provided support for subspecies recognition, showing that *G*. *a*. *arenarius* along the Rio Grande River valley were larger in size than *G*. *a*. *brevirostris* in White Sands. However, their results also led them to recognize specimens from Socorro Co. (north of White Sands) as belonging to *G*. *bursarius* showing that morphological characteristics are not always reliable in defining taxonomic boundaries in this genus. Hafner and Geluso (1983), using allozymic and karyotypic data, synonymized *G*. *arenarius* under *G*. *bursarius* based on a lack of fixed allelic differences and an interpretation of allele frequencies and karyotype as reflecting gene flow between the two taxa. Despite the synonomy, they maintained the subspecies taxonomy of Hall (1932), adding the populations in Socorro Co. to the subspecies *G*. *b*. *brevirostris* and classifying specimens from Fort Sumner as *G*. *b*. *knoxjonesi* (currently recognized as *G*. *knoxjonesi*; Wilson & Reeder, 2005). Most recently, mitochondrial and nuclear data placed *G*. *arenarius* as a sister taxon to *G*. *knoxjonesi* within the *G*. *bursarius* species group (Chambers et al., 2009; Sudman et al., 2006). Currently, the distribution of the subspecies *G*. *a*. *arenarius* (Figure 1) is considered to include the vicinity of Samalayuca, Mexico, the Rio Grande River valley of northern Chihuahua, Mexico and western Texas (El Paso and Hudspeth counties) to Las Cruces, NM (Doña Ana Co.) west toward Deming, NM (Luna Co.). North of *G*. *a*. *arenarius*, the distribution of the subspecies *G*. *a*. *brevirostris* is considered to include three documented locations (Figure 1): the vicinity of White Sands National Monument in the Tularosa Basin (Otero Co.); near San Antonio, NM northeast of the Jornada del Muerto Basin (Socorro Co.); and near Gran Quivira National Monument (Socorro Co.).

The goal of our study was to document the geographic patterns of population genetic diversity of *G*. *arenarius* using nuclear and mitochondrial data. Our specific objectives were to (1) evaluate the correspondence of genetic patterns to the traditionally accepted subspecies boundaries, (2) map the distribution of potentially suitable soils to identify barriers or corridors to dispersal and to guide future survey efforts, and (3) to inform conservation priorities by providing evidence that could be used to recognize distinct population segments (the smallest division of a taxonomic species permitted to be protected under the U.S. Endangered Species Act) and by quantifying the relative degree of genetic diversity within populations.

## 2 MATERIALS AND METHODS

### 2.1 Sample collection and DNA extraction

Samples were obtained by trapping using Macabee and Victor gopher traps and from tissue loans from the New Mexico Museum of Natural History and Science (specimens have since been transferred to the Southwestern Museum of Biology). Tissues from specimens collected near the Samalayuca dunes were imported into the United States by approval from the USFWS (#2021ME2698399). Trapping was conducted following the American Society of Mammalogists guidelines (Sikes et al., 2016). A total of 74 specimens of *G*. *arenarius* were obtained representing almost all of the known populations of both subspecies (Appendix S1; Figure 1). Tissue from one *G*. *knoxjonesi*, the sister taxon to *G*. *arenarius*, was also obtained for use as an outgroup in some analyses. DNA was extracted from liver, spleen, or muscle tissue by phenol extraction or using DNeasy Blood and Tissue kits (QIAGEN).

The only known populations not represented in our dataset are those from Luna Co., NM (gophers at this location have not been documented since their original discovery in 1889 despite repeated attempts by a colleague, D. Hafner, pers. comm.), those from near the U.S./Mexico border (which apparently have not been documented since 1959), and those from western Doña Ana Co., NM (which is only 22 km from the nearest sampled locality).

### 2.2 AFLP methodology

The AFLP protocol was modified from Vos et al. (1995). Fifty nanograms of total genomic DNA was digested for 3 h at 37°C with 20 units of *Ase*I (New England Biolabs), 20 units of *EcoR*I (New England Biolabs), and 1× restriction enzyme buffer. Ligations were performed by adding 75 pmoles each of two double‐stranded adapters *EcoR*I (5′‐AATTGGTACGCAGTCTAC‐3′/5′‐CTCGTAGACTGCGTACC‐3′) and *Ase*I (5′‐TACTCAGGACTCAT‐3′/5′‐AGTCCTGAGTAGCAG‐3′), 4 μL of 10× ligation buffer, 3 units T4 DNA ligase (New England Biolabs), and 12 μL of H_2_O to restriction digestion products and incubating for 16 h at 160°C. Ligation products were diluted by adding 160 μL of 10 mM Tris (pH 8.5). A subset of ligated fragments was amplified by polymerase chain reaction (PCR) using preselective primers (*EcoR*I‐C 5′‐ACTGCGTACCAATTCC‐3′; AseI‐T 5′‐GATGAGTCCTGAGTAATT‐3′). Amplifications were carried out in 50‐μL reaction volumes containing 10 μL of diluted ligation product, 0.15 μM of both preselective primers, 1× buffer, 1.5 mM MgCl_2_, 0.8 mM deoxynucleotide triphosphates, and 2.5 units of GoTaq® DNA polymerase (Promega). Amplification conditions included an initial step of 72°C for 60 s followed by 20 cycles of 94°C for 50 s, 56°C for 60 s, and 72°C for 120 s. Five microliters of the preselective PCR products were diluted in 90 μL of 10 mM Tris (pH 8.5) and used as the template for the selective PCRs. A total of 25 selective primer pairs (which extended an additional two base pairs beyond the 3′ end of the preselective primers) were tested on a subset of individuals prior to selecting eight pairs for use with all individuals. These included EcoRI‐CAC paired with AseI‐TAT, TCA, TCC, TCT, TGA, TGG, TGT, and TTC. The following criteria were used in the selection of these eight primer pairs: the production of clearly discernable 70–400 bp fragments with a distribution of approximately 1–5 fragments for every 20 bp (to minimize the chance of homoplasy) and an absence of over‐amplified fragments which tend to minimize the amplification of other fragments. Selective PCR amplifications were carried out in 25 μL reaction volumes containing 5 μL of diluted preselective product, 0.15 μM both selective primers, 1× buffer, 1.5 mM MgCl_2_, 0.8 mM deoxynucleotide triphosphates, and 1.25 units of Taq DNA polymerase. The thermal profile for selective reactions was as follows: 24 cycles of 94°C for 50 s, 65–56.6°C (0.70°C reduction for 2nd through 13th cycle) for 60 s, and 72°C for 120 s followed by 23 cycles of 94°C for 50 s, 56°C for 60 s, and 72°C for 120 s. The *EcoR*I primer used in the selective reactions was fluorescently labeled. Selective PCR products were visualized (with internal size standards) using a Beckman‐Coulter CEQ8000 Automated Genetic Analysis System (Beckman‐Coulter, Inc.). Only AFLP fragments that could be unambiguously scored as present or absent were included in the data set.

Population structure was assessed using principal coordinate analysis (PCoA) in GenAlEx 6.51 (Peakall & Smouse, 2006, 2012) and STRUCTURE 2.3.4 (Falush et al., 2007; Pritchard et al., 2000). In STRUCTURE, 10 runs were performed with 1,000,000 iterations and 100,000 discarded for the burn‐in. Values of K ranging from 1 to 8 were tested under a model of admixture and correlated allele frequencies. StrAuto (Chhatre & Emerson, 2017) which implements GNU parallel (Tange, 2023) was used to automate STRUCTURE runs across multiple cores of a Jetstream virtual machine (Stewart et al., 2015; Towns et al., 2014). Structure Harvester (Earl & vonHoldt, 2012) was used to assess likelihood values across multiple values of K. Results of multiple STRUCTURE runs at each value of K were visualized using CLUMPAK (Kopelman et al., 2015), which implements CLUMPP (Jakobsson & Rossenberg, 2007) and DISTRUCT (Rosenberg, 2004) to align runs across K values and identify major and minor modalities among runs (Jakobsson & Rossenberg, 2007). In addition to performing STRUCTURE runs for K = 1–8, a hierarchical STRUCTURE analysis was conducted by performing separate STRUCTURE runs for each of the two groups defined by K = 2 (the two groups corresponded to current subspecies designations). For all subsequent analyses, localities were combined into populations based on the patterns revealed by PCoA and STRUCTURE. Specifically, locations 4 and 5 within Doña Ana Co. and 6 and 7 within El Paso Co. (Figure 1) were combined because they did not exhibit signs of population subdivision at the level of collecting locality.

The software package SNAPP (Bryant et al., 2012), implemented within BEAST 2.1 (Bouckaert et al., 2014), was used to infer phylogenetic relationships among populations. SNAPP infers trees from biallelic markers by implementing a full multispecies coalescent model. Because it is computationally expensive, four individuals were randomly selected to represent each population (based on the groupings guided by PCoA and STRUCTURE). SNAPP was run using MCMC length = 1,000,000, preburn‐in = 1000, samplefreq = 1000 with default parameters for mutation rate, coalescent rate, and ancestral population sizes. TreeAnnotator was used to construct the maximum clade credibility tree and calculate posterior probabilities. Trees were visualized using DensiTree 2.2.7 (provided with BEAST) and FigTree 1.4.4 (http://tree.bio.ed.ac.uk/software/figtree/).

Pairwise Φ_PT_ values (between each population) were calculated in GenAlEx 6.51 (Peakall & Smouse, 2006, 2012). In the absence of barriers to dispersal, genetic distance can be positively correlated with geographic distance (isolation by distance). We performed a Mantel test (Mantel, 1967) implemented in GenAlEx 6.51 to test for a correlation between linearized versions of pairwise Φ_PT_ values (Φ_PT_ [Φ_PT_/(1 − Φ_PT_)]) and geographic distance. Genetic diversity within populations was measured using unbiased expected heterozygosity (H_e_, gene diversity) and proportion of polymorphic loci (%P) calculated using GenAlEx 6.51.

### 2.3 mtDNA methodology

A portion of the mitochondrial ND2 gene was amplified by PCR using the primers H6313 (5′‐CTCTTATTTAAGGCTTTGAAGGC‐3′; Johnson & Sorenson, 1998) and L5215 (5′‐TATCGGGCCCATACCCCGAAAAT‐3′; Hackett, 1996). PCR reactions were carried out in final volumes of 25 μL consisting of 1× buffer, 2.5 mM MgCl_2_, 0.16 mM each dNTP, 0.1 μM each primer, and 0.05 U Taq DNA polymerase (Qiagen). PCR reactions were performed as follows: initial denaturation at 95°C for 5 min followed by 40 cycles of 95°C for 1 min, 50°C for 30 s, 72°C for 1 min 30 s and a 72°C final extension for 10 min. PCR products were cleaned using ExoSAP‐IT (Affymetrics) and sequenced bi‐directionally, using Beckman‐Coulter chemistry, with the same forward and reverse primers as used for PCR. Sequencing products were cleaned by ethanol precipitation and visualized using a Beckman‐Coulter CEQ 8000 Genetic Analysis System. Sequences were aligned to a reference and visually inspected for errors and low‐quality base calls using Beckman‐Coulter software.

A median‐joining haplotype network was created using POPART 1.7 (Bandelt et al., 1999; Leigh & Bryant, 2015). A phylogenetic tree was constructed from sequences of individuals using MrBayes 3.2.7a (Ronquist et al., 2012) and included the sister species *G*. *knoxjonesi* as an outgroup. The best‐fit substitution model was determined to be HKY + G in jModelTest v.2.1.10 (Darriba et al., 2012; Guindon & Gascuel, 2003) using BIC. Sample and print frequencies were set to 500, the diagnostic frequency was 5000, and the run length was 1,000,000. Trees were summarized to produce posterior probabilities of each split and branch lengths. The resulting tree was visualized using FigTree 1.4.4 (http://tree.bio.ed.ac.uk/software/figtree/). Genetic distances (uncorrected p‐distance and Kimura 2‐parameter) between subspecies and between *G*. *arenarius* and its sister species *G*. *knoxjonesi* were computed using the program MEGA‐X (Kumar et al., 2018).

Measures of mitochondrial genetic diversity and historical demography were made using Arlequin 3.5.2.2 (Excoffier & Lischer, 2010). These included haplotype diversity, nucleotide diversity, number of polymorphic sites, Fu's Fs, Tajima's *D*, and mismatch distribution analysis. The raggedness index of Harpending (1994) and the sum of squared deviations were employed to test the goodness of fit of the observed mismatch distribution to that expected under the model of sudden demographic expansion.

### 2.4 Distribution of potentially suitable soils

Soil classification maps were created from the Soil Survey Geographic Database (SSURGO; https://sdmdataaccess.sc.egov.usda.gov) using QGIS (QGIS Development Team, 2021). Soil survey data containing detailed descriptions of soil type and depth were only available for the United States. Soil characteristics were determined from USDA soil surveys (Bourlier & Neher, 1970; Bulloch & Neher, 1980; Cates & White, 2017; Derr, 1981; Jaco, 1971; Johnson, 1988; Neher, 1984; Neher & Bailey, 1976; Neher & Buchanan, 1980; Sprankle, 1983, 2004). Locations of known occurrences of *G*. *arenarius* (Table S1) also were plotted. These included only those locations which had geographic coordinates or exact locality data recorded by the collector and excluded records that only documented locations based on distance from a landmark (such as a city). Based on these known occurrences, soil types inhabited by *G*. *arenarius* were identified. Lastly, soils with characteristics known to support populations of *Geomys* (specifically, soils that were classified as sandy, sandy loam, or loamy sand to a depth of at least 12 inches) were identified and mapped using QGIS.

## 3 RESULTS

### 3.1 AFLP analyses

A total of 275 AFLP fragments (putative loci) were included in the final dataset of 74 individuals from eight sampled locations representing both subspecies. Of the 275 putative loci, 119 (43.3%) were polymorphic across the 74 individuals.

**FIGURE 2:**
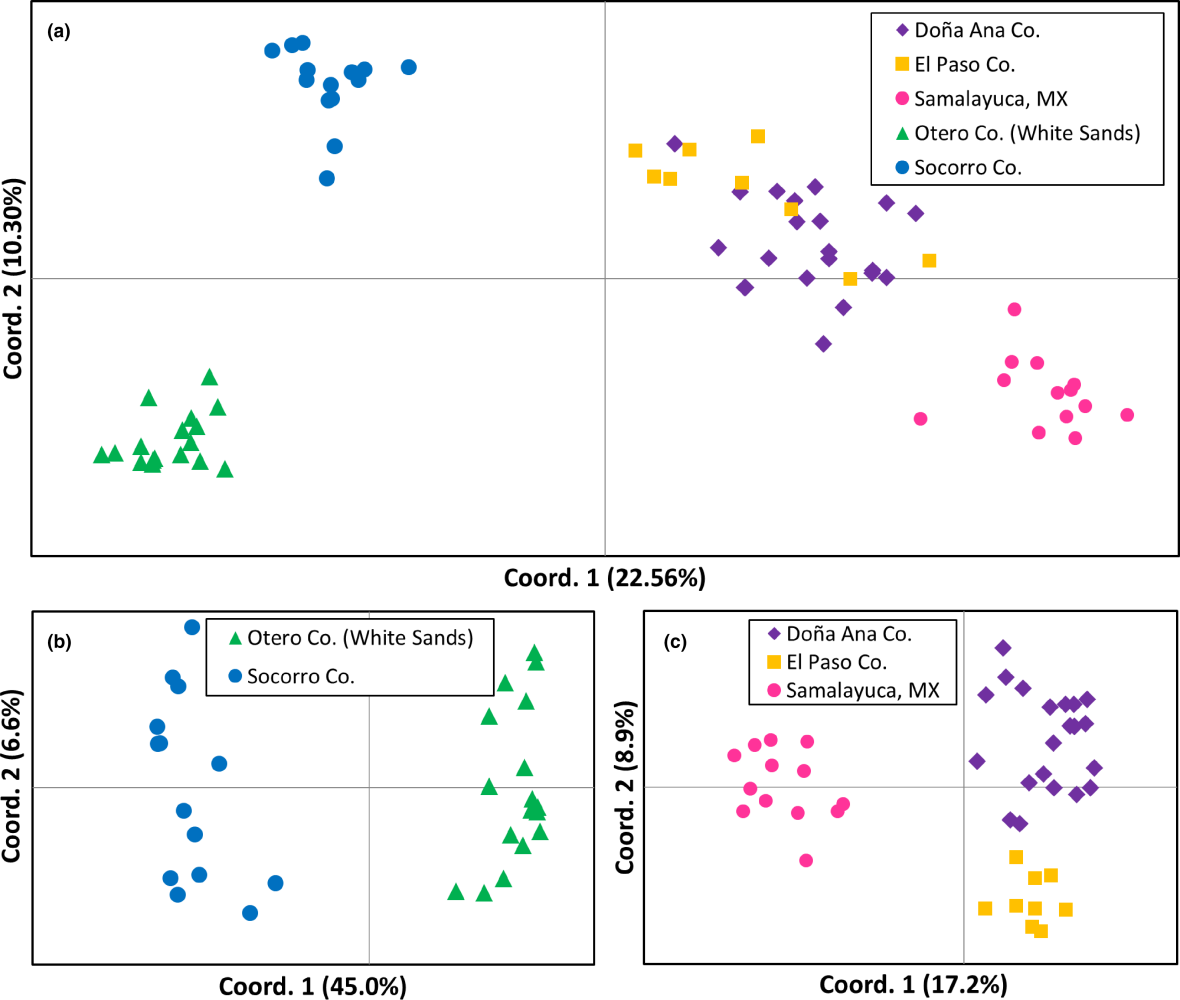
Principal coordinate analyses based on AFLPs of (a) *Geomys arenarius* (both subspecies), (b) *G. a. brevirostris*, and (c) *G. a. arenarius*. The proportion of variation explained by each axis is shown.

The PCoA for the entire dataset (Figure 2a) showed two distinct groups separating on axis 1 (corresponding to the two subspecies) and revealed further subdivision within subspecies when investigated by performing PCoA on each subspecies separately. PCoA performed on *G*. *a*. *brevirostris* showed two clusters corresponding to specimens from White Sands and Socorro Co. (Figure 2b). PCoA performed on *G*. *a*. *arenarius* showed three clusters, with the Samalayuca, MX specimens separated from those of the Rio Grande River (Doña Ana and El Paso counties) along axis 1 and specimens from Doña Ana and El Paso counties separated on axis 2 (Figure 2c).

**FIGURE 3:**
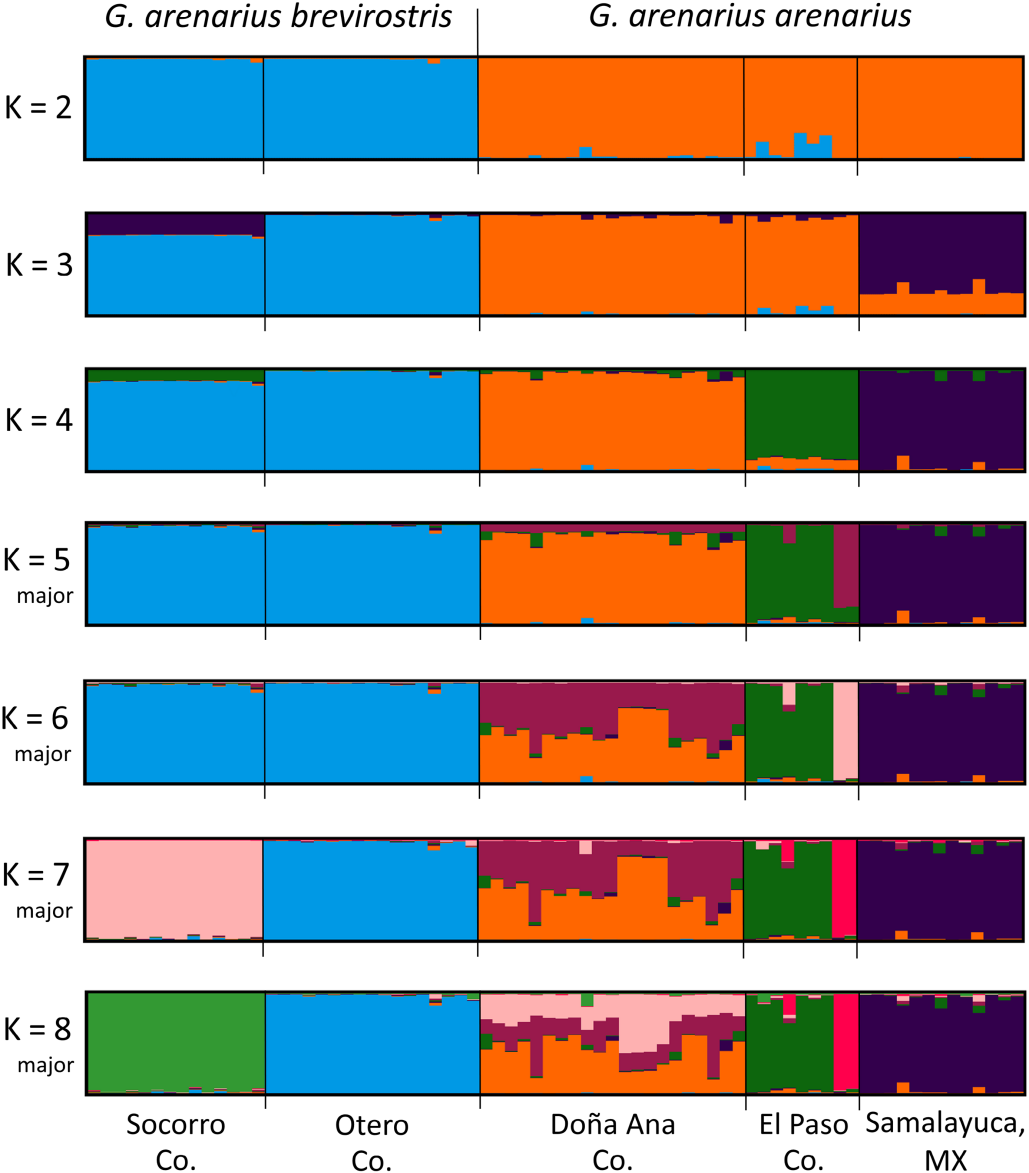
Results of STRUCTURE analyses based on AFLPs of *Geomys arenarius* for K = 2–8. Bars in each graph show membership coefficients. For instances when there were major and minor modalities, major modalities are shown.

STRUCTURE analyses showed individuals grouping by subspecies for K = 2 with further subdivision for values of K = 3–8 (Figure 3). With one exception, these groupings corresponded to collecting locality and were concordant with PCoA clusters. The exception was two individuals within El Paso Co. which were assigned to a separate group for K > 5 even though other individuals not in that group were collected from the same locality. This grouping appears to reflect subtle patterns in the data unrelated to geographic structure (perhaps a close familial relationship). Multimodality, the occurrence of more than one distinct clustering outcome among multiple runs (Jakobsson & Rossenberg, 2007), was present for values of K = 5, 6, 7, and 8 (Figure S1). In each instance, the minor modality of smaller values of K reflected patterns revealed by larger values of K and, with the one exception mentioned previously, were consistent with geographical sampling and PCoA clusters. The K = 5 minor modality best reflected the geographical sampling of individuals and clustering in the PCoA. A hierarchical STRUCTURE analysis, in which runs were performed using data for each subspecies separately, revealed the same patterns as described above (Figure S2).

**FIGURE 4:**
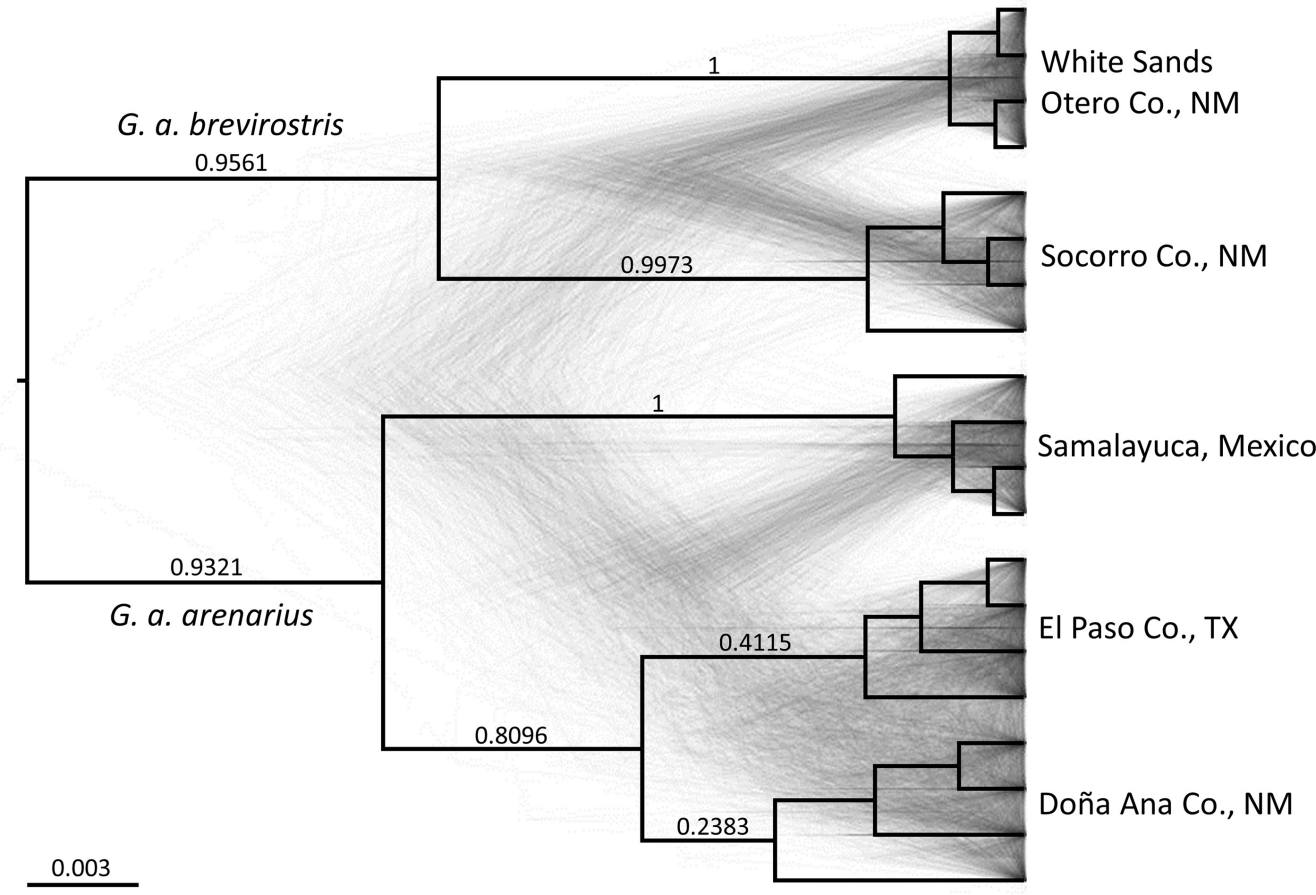
SNAPP phylogenies based on AFLPs of *Geomys arenarius* showing the complete set of consensus trees (gray) and the maximum clade credibility tree (black) with posterior probabilities.

The phylogenetic reconstructions inferred from analysis of AFLP data using SNAPP (Figure 4) were concordant with the population structure inferred from PCoA and STRUCTURE. Specifically, the greatest divergence was between subspecies with further divergence occurring between populations within subspecies. The Doña Ana and El Paso county populations were not well separated in the SNAPP phylogeny and had low posterior support values (<0.5).

Pairwise Φ_PT_ ranged from 0.170 between Doña Ana and El Paso counties to 0.658 between White Sands and Samalayuca, MX (Table 1). There was no significant correlation between pairwise Φ_PT_ and geographic distance (*R* ^2^ = 0.1425; *p* = .210). Unbiased expected heterozygosity ranged from 0.027 (White Sands) to 0.083 (Doña Ana Co.) and the proportion of polymorphic loci ranged from 8.4% (White Sands) to 28.7% (Doña Ana Co.; Table 2).

**TABLE 1:** Pairwise Φ_PT_ values based on AFLP data (below the diagonal) between populations of *Geomys arenarius* as defined by PCoA and STRUCTURE.

	*G*. *a*. *arenarius*			*G*. *a*. *brevirostris*	
	Doña Ana Co.	El Paso Co.	Samalayuca, MX	Otero Co. (White Sands)	Socorro Co.
Doña Ana Co.		.001	.001	.001	.001
El Paso Co.	.170		.001	.001	.001
Samalayuca, MX	.301	.363		.001	.001
Otero Co. (White Sands)	.469	.588	.658		.001
Socorro Co.	.399	.450	.592	.584	

*Note*: *p*‐Values (above the diagonal) are derived from 999 permutations (the smallest *p*‐value reported by GenAlEx is .001).

**TABLE 2:** Number of *Geomys arenarius* individuals with AFLP data (*n*) and AFLP genetic diversity of *G*. *arenarius* populations as defined by PCoA and STRUCTURE including unbiased expected heterozygosity (H_e_) and proportion of polymorphic loci (%P).

	*n*	H_e_ (SE)	%P
*G*. *a*. *arenarius*			
Doña Ana Co.	21	0.083 (0.009)	28.7
El Paso Co.	9	0.052 (0.008)	17.8
Samalayuca, MX	13	0.058 (0.008)	19.3
*G*. *a*. *brevirostris*			
Otero Co. (White Sands)	17	0.027 (0.006)	8.4
Socorro Co.	14	0.038 (0.008)	9.8

### 3.2 mtDNA analyses

ND2 sequences (trimmed to omit missing data at each end) were 828 bp long and obtained from 64 of the 74 individuals of *G*. *arenarius* and one *G*. *knoxjonesi* (for use as an outgroup). GenBank numbers (MW558503–MW558567) are provided for each specimen in Appendix S1. No indels or premature stop codons were observed. Among all individuals of *G*. *arenarius*, there were 21 haplotypes with 69 polymorphic sites, 57 transitions, and 12 transversions. Diversity measures for subspecies, populations within subspecies (as defined by AFLP analyses), and overall, are given in Table 3. All measures of genetic diversity were lower for *G*. *a*. *brevirostris* relative to *G*. *a*. *arenarius*. Compared to all other populations, the Samalayuca, MX population had the highest nucleotide diversity, greatest number of polymorphic sites, and second highest haplotype diversity (El Paso Co. had a slightly higher haplotype diversity). The White Sands population contained the fewest number of haplotypes, lowest haplotype diversity, lowest number of polymorphic sites, and lowest nucleotide diversity compared to all other populations.

**TABLE 3:** Genetic diversity of *Geomys arenarius* (partitioned by subspecies and population) based on mtDNA ND2 sequences: number of individuals with ND2 mtDNA sequences (*n*), number of haplotypes (*n* ^hap^), haplotype diversity (*h*), nucleotide diversity (*π*), number of polymorphic sites (PS), Fu's Fs (Fs), Tajima's *D* (*D*), Raggedness index (Rg), sum of squared deviations (SSD), and *p*‐values of statistical tests (*p*).

Subspecies and population	*n*	*n* ^hap^	*h* (±SD)	*π* (±SD)	PS	Fs (*p*)	*D* (*p*)	Rg (*p*)	SSD (*p*)
*G*. *a*. *arenarius*	39	14	0.912 (0.0254)	0.0096 (0.005047)	31	0.5733 (.62700)	0.2760 (.69600)	0.0240 (.1640)	0.0201 (.0040)
Doña Ana Co., NM	18	4	0.680 (0.0795)	0.0037 (0.002234)	9	2.9690 (.91700)	0.5546 (.73600)		
El Paso Co., TX	8	5	0.893 (0.0858)	0.0034 (0.002285)	7	−0.4139 (.33700)	0.2145 (.60100)		
Samalayuca, MX	13	5	0.833 (0.0597)	0.0081 (0.004616)	15	3.5386 (.93800)	1.6504 (.97300)		
*G*. *a*. *brevirostris*	25	7	0.753 (0.0669)	0.0045 (0.002612)	12	1.2084 (.75400)	0.5724 (.75600)	0.1480 (.0840)	0.0582 (.1130)
Otero Co., NM (White Sands)	12	2	0.167 (0.1343)	0.0002 (0.000327)	1	−0.4757 (.13100)	−1.1405 (.17000)		
Socorro Co., NM	13	5	0.756 (0.0974)	0.0022 (0.001519)	6	−0.2912 (.41100)	−0.2158 (.46700)		
Overall	64	21	0.931 (0.0150)	0.0277 (0.013707)	69				

**FIGURE 5:**
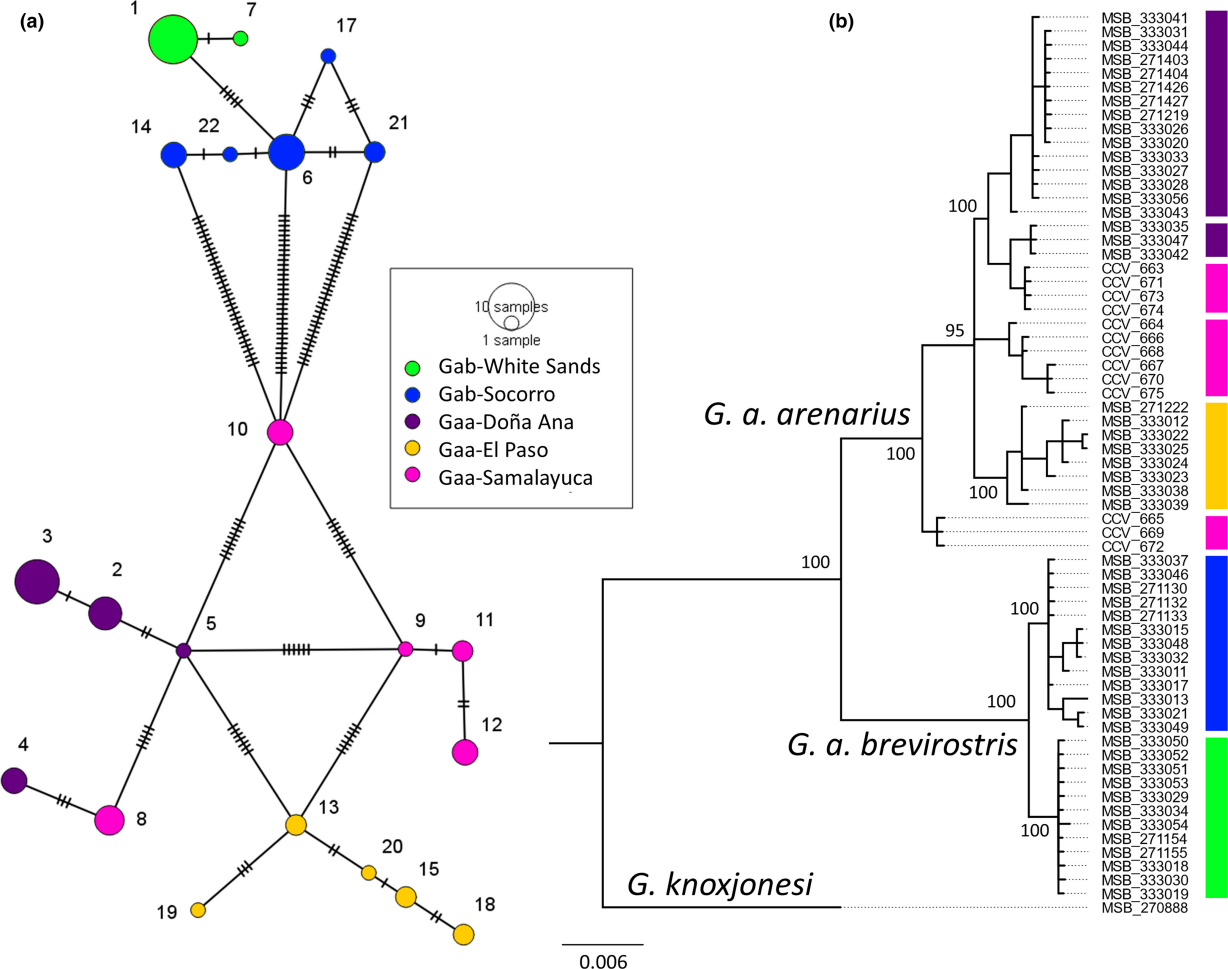
Median‐joining network (a) and Bayesian phylogenetic tree of individuals (b) of *Geomys arenarius* based on ND2 mtDNA DNA sequences. Size of circles in the network are proportional to haplotype frequency and lines across branches represent mutational steps between haplotypes. The color of circles in the network indicates population. Haplotype numbers correspond to those in Appendix S1. Numbers along branches in the phylogenetic tree indicate support (posterior probability; provided only for major clades) and colored bars correspond to populations as in the haplotype network.

The median‐joining network of ND2 haplotypes (Figure 5a) showed that all haplotypes were restricted to sampling locations—no haplotypes were shared among subspecies or populations within subspecies. Within most sampling locations, haplotypes were separated by relatively few (1–3) mutational steps, with one notable exception—the Samalayuca, MX population contained haplotypes differing by a much greater number of mutational steps.

The ND2 phylogenetic tree showed individuals representative of subspecies forming highly supported reciprocally monophyletic clades (Figure 5b). The two populations of *G*. *a*. *brevirostris* formed highly supported reciprocally monophyletic clades. Genetic distance between subspecies (both uncorrected p‐distance and K2P distances) was 5%. For comparison, genetic distance between *G*. *arenarius* and its sister species *G*. *knoxjonesi* was 9%.

**FIGURE 6:**
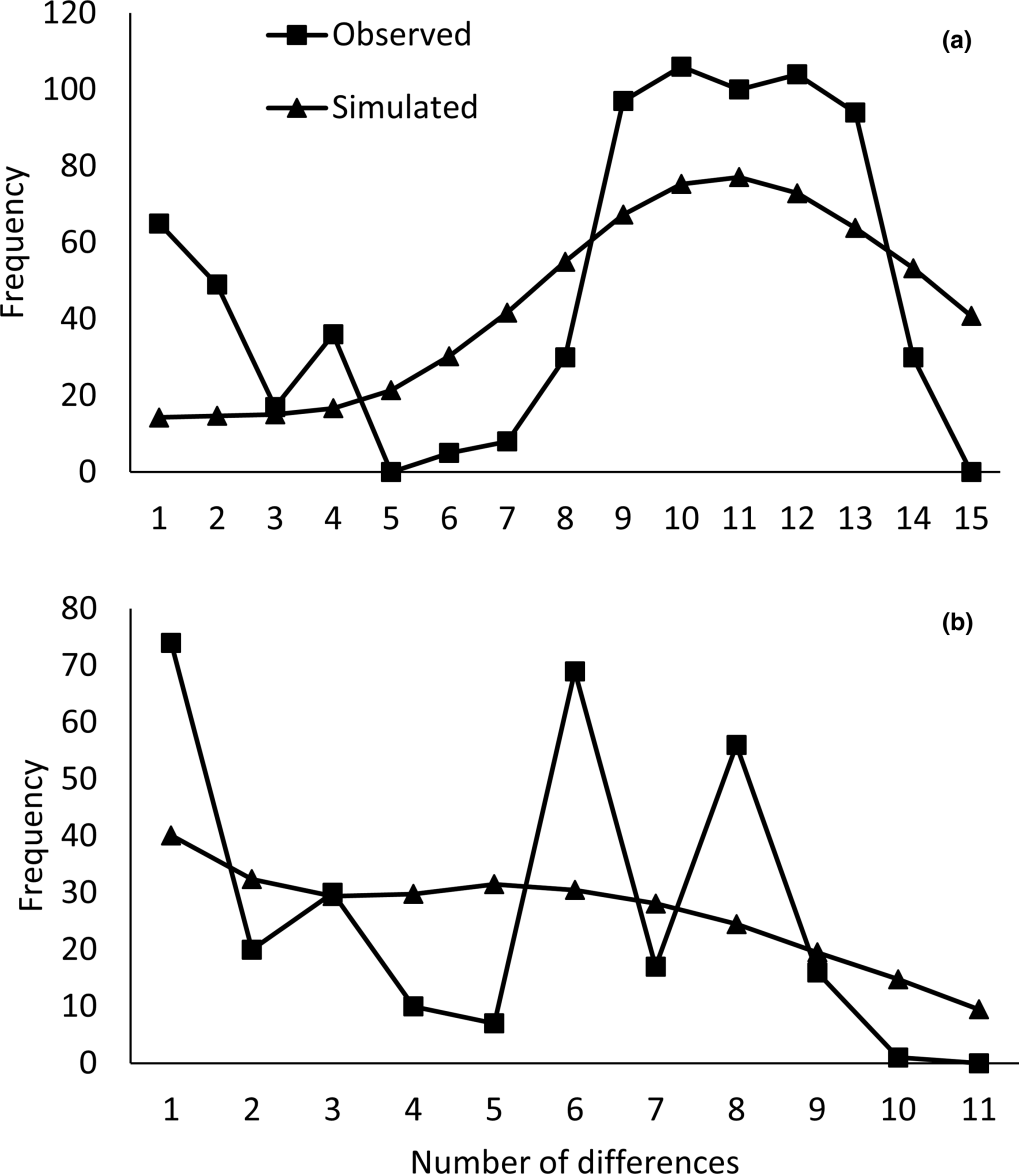
Mismatch distribution for *G. a. arenarius* (a) and *G. a. brevirostris* (b) showing the observed and expected distribution of pairwise differences among ND2 haplotypes under a demographic expansion model.

Tajima and Fu's statistics were not significantly negative (*p* > .05; Table 3) which suggest the absence of demographic expansion. At the subspecies level, the mismatch distribution for *G*. *a*. *arenarius* appeared multimodal (Figure 6; multimodal distributions indicate the absence of recent demographic expansion). SSD showed a significant deviation (*p* = .004) from the distribution expected under population expansion (Table 3) but Harpending's raggedness index (Rg) was not significantly different (*p* > .05). For *G*. *a*. *brevirostris*, the mismatch distribution was clearly multimodal, but both Rg and SSD were nonsignificant (*p* > .05) indicating a good fit to the model of population expansion. For these two indices (Rg and SSD), calculations were not performed at the level of sampling localities because sample sizes were insufficient for most populations.

### 3.3 Distribution of potentially suitable soils

Soil types associated with each locality of known occurrence are reported in Table S1. In Socorro Co., NM, soils inhabited by *G*. *arenarius* were classified as Mespun fine sand. In Otero Co., NM (White Sands) inhabited soils included the Lark‐Transformer and Astrobee‐Lark association (consisting of deep, gypsiferous sand and sandy loam soils). In Doña Ana Co., NM, inhabited soils included Brazito loamy fine sand, Brazito very fine sandy loam, Anthony‐Vinton fine sandy loams, one occurrence in Glendale loam, and one occurrence in Agua clay loam (but immediately adjacent to Brazito very fine sandy loam). In El Paso Co., TX, inhabited soils included Gila fine sandy loam, Harkey loam (which consists of loamy very fine sand, fine sandy loam, loam, and very fine sandy loam), Made Land (Gila soil material which consists of silty clay loam, fine sandy loam, and sand which has been modified by human activity), and Saneli silty clay (adjacent to Vinton fine sandy loam and Brazito loamy fine sand). In addition to the locations in El Paso Co., TX which had accurate coordinates, locations southeast of Fabens had recorded coordinates that were inaccurate—however, we know these specimens were collected adjacent to an irrigation ditch that parallels Texas Highway 20. Soils in this area are classified as Glendale silty clay loam, Tigua silty clay, Glendale loam, or Harkey silty clay loam. The soils classified as clay loam included smaller areas of Glendale loam, Harkey loam, and Gila loam (Jaco, 1971) and have been modified by human activity in association with irrigation. Additionally, these locations were within 500 m of soils classified as Harkey loam and Brazito very fine sandy loam.

Based on USDA soil surveys and SSURGO data, soils that we identified as potentially habitable by *G*. *arenarius* were discontinuous and not fully occupied based on currently documented occurrence data (Figure 1). Unsuitable soils appeared to separate the distribution of *G*. *a*. *brevirostris* into two habitable areas (one in White Sands and one in Socorro Co.). In contrast, potentially suitable soils appeared to be relatively continuous within the distribution of *G*. *a*. *arenarius* for which soils data were available.

## 4 DISCUSSION

### 4.1 Population structure and taxonomy

Our study documents the phylogeographic and population genetic structure among most known populations of *G*. *arenarius*, providing support for the geographic boundaries of the recognized subspecies and revealing further genetic subdivision within each subspecies. These patterns were reflected in both the nuclear AFLP and mitochondrial ND2 datasets. Additionally, the three approaches toward analyzing the AFLP data (PCoA, STRUCTURE, and SNAPP), each based on differing algorithms and assumptions, consistently identified the same patterns of hierarchical clustering. The greatest genetic divergence was between populations of Socorro Co. and White Sands to the north (representing *G*. *a*. *brevirostris*) and populations of Doña Ana and El Paso counties and Samalayuca, MX to the south (representing *G*. *a*. *arenarius*)—this pattern was consistent with currently recognized subspecies boundaries.

Determining an appropriate taxonomic level at which to recognize genetically distinct, allopatric populations is largely subjective because of the nature of evolutionary divergence and subsequent lack of universally accepted definitions or concepts for these entities. Genetic and phenotypic divergence among populations often occurs along a continuum, parts of which can be recognized as distinct population segments and, more formally, species (or subspecies) which can be supported by various lines of evidence (De Queiroz, 2007; Frankham et al., 2012). Below the level of species, mammalian subspecies have traditionally required, by most workers, to be recognizable entities (morphologically or genetically) that are separated geographically (Lidicker, 1962; Patten, 2010; Taylor, Archer, et al., 2017; Taylor, Perrin, et al., 2017; Wilson & Brown Jr, 1953). However, some organisms (such as geomyids) can easily become isolated into allopatric populations with very small effective population sizes and quickly diverge in allele frequencies to become recognizable as distinct entities.

Both nuclear and mitochondrial datasets showed further subdivision within each subspecies. Within *G*. *a*. *brevirostris*, the samples from two locations within Socorro Co. formed a single genetically defined population separate from those of White Sands. Within the other subspecies, *G*. *a*. *arenarius*, specimens from El Paso and Doña Ana counties were most similar (with only minimal divergence between them) but were distinct from the Samalayuca, MX samples. Although both AFLP and mtDNA data revealed five genetically distinct populations, the patterns differed between the two subspecies. Within *G*. *a*. *brevirostris*, the two AFLP‐defined populations were reciprocally monophyletic in the mtDNA phylogeny. Within *G*. *a*. *arenarius*, the three AFLP‐defined populations were not reciprocally monophyletic in the mtDNA tree but were nonetheless genetically distinct based on a complete lack of shared mtDNA haplotypes.

Hafner and Geluso (1983) interpreted their allozyme allele frequency data of *G*. *arenarius* as evidence of clinal variation from northern‐most to southernmost locations, suggestive of gene flow between nearby *G*. *knoxjonesi* and *G*. *arenarius*. However, this interpretation appears to conflict with their reported genetic similarity coefficients which show the *G*. *knoxjonesi* population at Fort Sumner to be *least* similar to the adjacent Socorro Co. population of *G*. *arenarius*. We view their allozyme data as being compatible with genetic drift acting on shared ancestral polymorphisms rather than evidence of clinal variation which would require ongoing gene flow among populations that appear to be disjunct. Our data indicated that the White Sands and Socorro Co. populations are allopatric and most closely related to one another relative to other populations. This pattern of relationship is consistent with figure 3 of Hafner and Geluso (1983; a phenogram based on allozyme data). The lack of a significant pattern of isolation by distance across the sampled distribution of *G*. *arenarius*, the geographically restricted mtDNA haplotypes, and the discontinuous distribution of potentially suitable soils supports the view that population structure within *G*. *arenarius* is driven largely by genetic drift within geographically isolated populations rather than clinal variation.

### 4.2 Factors impacting distribution

The origin of geographically isolated populations can be explained by either dispersal or vicariance (Nelson & Platnick, 1981; Udvardy, 1969). Populations resulting from dispersal are those that were founded by members of an ancestral population which were able to move across preexisting barriers to establish new populations in an area beyond the ancestral distribution. In contrast, populations resulting from vicariance are those that were established when an ancestral population was geographically subdivided by formation of a barrier within the existing distribution. Because geomyids are considered to be a low‐dispersal species (Elrod et al., 2000), vicariance seems the most likely explanation for the geographically isolated populations of *G*. *arenarius*. While aboveground dispersal in *Geomys* has been documented, the distance traveled aboveground is almost certainly insufficient to traverse the unsuitable soils that appear to be separating subspecies and populations within subspecies. Connior (2008) documented a single 165 m aboveground dispersal of *G*. *bursarius* in Arkansas, and a dispersal of 147 m was documented for a translocated *G*. *pinetis* in Florida (Pynne et al., 2019). A dispersal event of 319 m was reported by Warren et al. (2017) for *G*. *pinetis* in Georgia. Panich (2006) did not detect any aboveground movement of *G*. *bursarius* during the course of their study in Wisconsin. Aboveground dispersal distances in *Geomys* are limited by their awkward locomotion due to musculoskeletal adaptations for burrowing and increased risk of predation when out of their burrows. The occurrence of unsuitable soils which separate populations of *G*. *arenarius* explains the apparently discontinuous distribution of this species and patterns of genetic divergence. Because soil distributions are dynamic over time, currently isolated populations of *G*. *arenarius* could be the product of soil loss fragmenting a formerly more widely distributed species. Changes in soil distribution are the result of soil deposition and deflation which occur in response to climate oscillations, orogeny, volcanism, and alternations in river flow, all of which are known to have occurred during the Neogene and Pleistocene periods within the region currently occupied by *G*. *arenarius*.

Southern New Mexico and northern Mexico has a long history of soil deposition and deflation. For example, the Strauss sand sheet (Figure 1) extends west and south of the Rio Grande River and formed during three phases of eolian deflation and deposition beginning 45 ka and continuing into modern times (Hall & Goble, 2015). *G*. *arenarius* is known to inhabit at least some portions of the Strauss sand sheet. During the Pleistocene, a large pluvial lake is thought to have existed in what is now the Strauss sand sheet (Wilson & Pitts, 2010), and *G*. *arenarius* would have been excluded from that area during this time. To the east of the Rio Grande River, the Bolson sand sheet (Figure 1) extends from just south of White Sands in Otero Co., NM through El Paso Co., TX but is not currently known to support populations of *G*. *arenarius*. The age of the Bolson sand sheet ranges from 45 to 22 ka (Hall et al., 2010). Complicating matters further, the soils and vegetative communities of southeastern New Mexico are different than they were before the arrival of Europeans and subsequent overgrazing by cattle. The historical desert grasslands of this area are now replaced by brushland and coppice dunes formed when blown sand collects around vegetation (Grover & Musick, 1990; Langford, 2000). Therefore, the occurrence of *G*. *arenarius* in modern times may not reflect their occurrence prior to the arrival of Europeans.

Within the Rio Grande river valley there exists an almost continuous distribution of soils with high sand content, and sampled populations within this region (locations 4, 5, 6, and 7; Figure 1) exhibited minimal genetic divergence relative to other sampled populations. It appears that the continuously distributed nature of suitable soils in this region has facilitated gene flow. The only potential barrier to gene flow that we identified within this area was a narrowing of the Rio Grande River valley between the Franklin Mountains on the north side of the river and higher elevations on the south side. Within this narrow gap, the soils immediately adjacent to the river channel consist of the Delnorte‐Cuntio Association—shallow gravelly loam over caliche or gravelly sandy loams (Jaco, 1971)— soils likely to be unsuitable for *G*. *arenarius*. Although we detected subtle genetic divergence between sampled locations on either side of this potential barrier, many additional sampled locations (with large sample sizes each) would be needed in order to distinguish between isolation by distance and reduced of gene flow due to the potential barrier itself.

In contrast to locations along the Rio Grande river valley, the Samalyuca, MX location exhibited relatively greater genetic divergence. Although the nearest documented population is only 40 km northeast along the Rio Grande river, it is possible that gene flow may have been to the northwest of Samalayuca via the Strauss sand sheet. The vicinity of Samalayuca, MX represents the southernmost extent of the Strauss sand sheet which extends northward to the vicinity of Las Cruces, NM (Hall & Goble, 2015) and may have connected the Samalayuca population to those currently inhabiting the northern portion of the Strauss sand sheet of Doña Ana Co. Specimens of *G*. *arenarius* have not been collected from the Strauss sand sheet in Mexico north of Samalayuca, but much of this area is difficult to access and few, if any, collecting efforts may have been attempted. Because of the insufficient knowledge regarding their occurrence north of Samalayuca, an understanding of connectivity of these populations relative to those along the Rio Grande and Samalayuca remains unknown.

The close relationship of the White Sands population with those to the north in Socorro Co. (relative to those to the south along the Rio Grande river) is challenging to explain. The Rio Grande river of New Mexico did not drain into the Gulf of Mexico until approximately 800,000 years ago when it joined the Pecos River. Prior to this, the Rio Grande emptied into closed basins (having no external drainage) which were formed by the opening of the Rio Grande rift 35 million years ago. Over time, the ancestral Rio Grande progressively integrated basins from north to south, eventually reaching Texas around 2 million years ago, at which time the river bifurcated and began flowing to the east side of the Franklin mountains—spilling through Fillmore Gap between the Organ and Franklin Mountains (Armour et al., 2018; Mack et al., 2006; Seager et al., 1984). The extent to which changes in the ancestral Rio Grande resulted in the deposition and erosion of soils habitable by *G*. *arenarius* (and, therefore, facilitating or blocking dispersal) is unknown, but it cannot be ruled out that the White Sands and Socorro Co. populations were once connected along the ancestral Rio Grande River valley west of the Oscura and San Andres mountains. Given the 5% mitochondrial divergence between the subspecies of *G*. *arenarius*, it is likely that at least some of these changes occurred during a time in which *G*. *arenarius* inhabited the region. We did not date the divergence between subspecies using mitochondrial DNA sequences given the uncertainty in mutational rate of geomyid rodents (Spradling et al., 2001) and paucity of clearly identifiable fossils for calibration dates.

Alternatively, the connection between White Sands and Socorro Co. populations may have been to the east of the Oscura Mountains. Within this area, these populations are separated by rugged terrain with unsuitable soils and a lava flow (the Carrizozo Malpais) which occurred only 5200 years ago (Dunbar, 1999), likely after the establishment of populations in White Sands and Socorro Co. However, unsuitable soils in this area may have prevented gene flow even in the absence of the Carrizozo Malpais. There is no evidence of rivers having flowed historically from north to south into the White Sands area, nor is there information available regarding historical soils that would have allowed connectivity between these populations east of the Oscura Mountains even in the absence of the Carrizozo Malpais. The terrain between localities 1 and 2 in Socorro Co. (separated by 64 km; Figure 1) is quite rugged with a patchwork of suitable and unsuitable soils, very unlike the landscapes in which other populations of *G*. *arenarius* occur. The lack of genetic divergence between these localities indicates that gene flow is ongoing (or occurred recently) despite the terrain. Given the apparent ability of *G*. *arenarius* to remain genetically connected over the terrain in this area, we cannot dismiss connectivity between the White Sands and Socorro Co. populations east of the Oscura Mountains during a time when soils within this area may have been somewhat more conducive to gene flow. The lack of data on the historical distribution soils precludes further evaluation of these alternate hypotheses.

Many of the areas with soils potentially suitable for *G*. *arenarius* are difficult to access and have not been surveyed, so it is unknown whether the apparent lack of occupancy in these potentially habitable areas is due to unsuitable soils or merely the lack of collecting efforts. Given the complexities of soil classification, the coarseness by which soils are mapped in certain areas, the uncertainty regarding which soil characteristics best define habitability by *G*. *arenarius*, and the potential for gophers to occupy unsurveyed areas, the resulting map of likely habitable soils should be interpreted with caution, but could be used to guide future distributional surveys.

### 4.3 Demographic history

Analyses of demographic history did not consistently support a model of demographic expansion within each subspecies as various test results were in conflict. The topology of the mitochondrial haplotype network did not fit the star pattern expected from recent population expansion where a common, shared haplotype is connected by numerous haplotypes separated by few mutational steps (Harpending et al., 1998; Slatkin & Hudson, 1991). In fact, there was a complete absence of mitochondrial haplotypes shared among populations of *G*. *arenarius*—a rarely documented phylogeographic pattern. If these subspecies had experienced recent population expansion, the haplotype network would not show such geographically restricted haplotypes. Overall, there was no strong support for population expansion within either subspecies; however, many sources of error and low statistical power can make the inference of demographic histories from genetic data challenging (Grant, 2015).

Many mammalian species have shown signatures of demographic expansion following the Last Glacial Maxima (LGM), including those in the southwestern United States and northern Mexico (Dragoo et al., 2006; Jezkova et al., 2015; Mantooth et al., 2013; Menchaca et al., 2020). Changes in environmental conditions and availability of suitable habitat before and after the LGM are likely causes of demographic contraction and expansion. *G*. *arenarius*, because it is almost exclusively subterranean, may be somewhat insulated from climatic changes and may not have experienced demographic expansion due to the protection of their more thermally stable underground environment (Pynne et al., 2021) and broad diet of monocots and dicots allowing for a continuous food source as plant communities change. Different patterns of genetic diversity and demographic history have been documented between species with different habitat requirements (e.g., *Dipodomys*; Jezkova et al., 2015), thus different phylogeographic patterns and demographic histories would be expected when comparing *G*. *arenarius* with other species inhabiting the Chihuahuan Desert, especially those that are not subterranean and have greater ability for dispersal in response to climatic changes.

### 4.4 Genetic diversity

All measures of genetic diversity (nuclear and mitochondrial) were lower within populations of *G*. *a*. *brevirostris* compared to those of *G*. *a*. *arenarius* indicating that populations of *G*. *a*. *bervirostris* have lower effective population sizes. Under the neutral model, genetic diversity depends on effective population size and mutation rate (Kimura, 1983). Given that mutation rate should be consistent across populations of *G*. *arenarius*, low genetic diversity would be the result of small population size or historical population reductions. Differences in population size could be caused by differences in the size of a habitable area or population density. Density, in turn, is likely impacted by food availability which has been shown for *G*. *arenarius* in the vicinity of Samalayuca dune fields to include both monocots and dicots in 10 plant families (Rueda‐Torres et al., 2022) and in the closely related species *G*. *bursarius* and *G*. *attwateri* to be primarily grasses and to a lesser extent forbs (Luce et al., 1980; Myers & Vaughan, 1965; Williams & Cameron, 1986). Little is known about the abundance of *G*. *arenarius* within Socorro Co., as they have been documented infrequently within this area, but based on our own observations, *G*. *arenarius* is abundant in the vicinity of White Sands. Despite their abundance, this population exhibited the lowest levels of genetic diversity suggesting a relatively recent bottleneck. During the late Pleistocene, the pluvial Lake Otero covered much, if not all, of the region currently occupied by *G*. *arenarius* (Allen et al., 2009) and may have greatly reduced the population size if *G*. *arenarius* occurred in this area during this time. Alternatively, this population could have been founded by a small number of individuals after Lake Otero receded and dune formation occurred.

Genetic diversity of the Samalayuca, MX population was higher than all other populations except Doña Ana Co., NM. This was unexpected given that *G*. *arenarius* has only been reported from the immediate vicinity of the Samalayuca dune field. The relatively high amount of genetic diversity of the Samalayuca, MX population suggests that *G*. *arenarius* may occupy (or have recently occupied) a much larger geographical area within Mexico than has been documented, perhaps within the Strauss sand sheet northwest of Samalayuca.

## 5 CONCLUSIONS

Our results show multiple levels of population subdivision within *G*. *arenarius*, reflecting the dynamic geomorphological history of the northern Chihuahuan Desert, particularly those processes resulting in deposition and deflation of soils with high sand content required by this species. The deepest pattern of genetic divergence coincided with the geographic boundaries of the recognized subspecies—*G*. *a*. *arenarius* and *G*. *a*. *brevirostris*. Genetic diversity varied considerably among populations due to differences in population size or unique demographic histories. Although most populations of *G*. *arenarius* do not appear to be threatened significantly by urbanization or agriculture, some populations may be negatively impacted by continued loss of desert grasslands due to grazing, conversion for agricultural use, or climate change. Of particular interest is the Samalayuca, MX population which is known only from the vicinity of the Samalayuca dune fields (Anderson, 1972; Fernández et al., 2014). This population contributes a substantial amount of genetic diversity to the species, and if the population is restricted to a small geographic area, could be impacted by human activity or climate change. However, the high level of genetic diversity suggests that this population may be much larger than is currently known. Efforts to survey for this species beyond the vicinity of the Samalayuca dune fields are needed to better understand the distribution of *G*. *arenarius* in Mexico.

## AUTHOR CONTRIBUTIONS

**Russell S. Pfau:** Data curation (lead); formal analysis (lead); investigation (lead); methodology (lead); project administration (lead); resources (equal); writing – original draft (lead). **Ana B. Gatica‐Colima:** Resources (supporting); writing – review and editing (supporting). **Philip S. Sudman:** Conceptualization (lead); investigation (equal); methodology (supporting); project administration (supporting); resources (equal); writing – original draft (supporting); writing – review and editing (supporting). **Ashley N. Kozora:** Investigation (equal); methodology (supporting); writing – original draft (supporting); writing – review and editing (supporting).

## FUNDING INFORMATION

This work was supported by Tarleton State University.

## Supporting information


Figures S1 and S2.

Appendix S1.

Table S1.

## Data Availability

DNA sequences are available on GenBank (MW558503–MW558567). Data openly available in a public repository that does not issue DOIs.
