# Supplementary material for: Population genetic structure of a Chihuahuan Desert endemic mammal, the desert pocket gopher, Geomys arenarius
Source: Ecol Evol. 2023 Sep 28;13(10):e10576. doi: 10.1002/ece3.10576 (PMC10539045; doi:10.1002/ece3.10576)
Supplement: Supplementary file 1 — Figures S1 and S2. [file ECE3-13-e10576-s001.pdf]

Supporting Information

Population genetic structure of a Chihuahuan Desert endemic, the desert pocket gopher, *Geomys arenarius*. Ecology and Evolution.

Russell S. Pfau[1*], Ashley N. Kozora[2], Ana B. Gatica-Colima[3], Philip S. Sudman[1]

[1] Department of Biological Sciences, Tarleton State University, Stephenville, Texas 76402, U.S.A., pfau@tarleton.edu
[2] Cooper High School, Abilene, Texas 79605, U.S.A., ashley.kozora@abileneisd.org
[3] Departamento de Ciencias Químico-Biológicas, Instituto de Ciencias Biomédicas, Universidad Autónoma de Ciudad Juárez, Anillo Envolvente del PRONAF y Estocolmo s/n, Ciudad Juárez, Chih. 32310, Mexico, agatica@uacj.mx

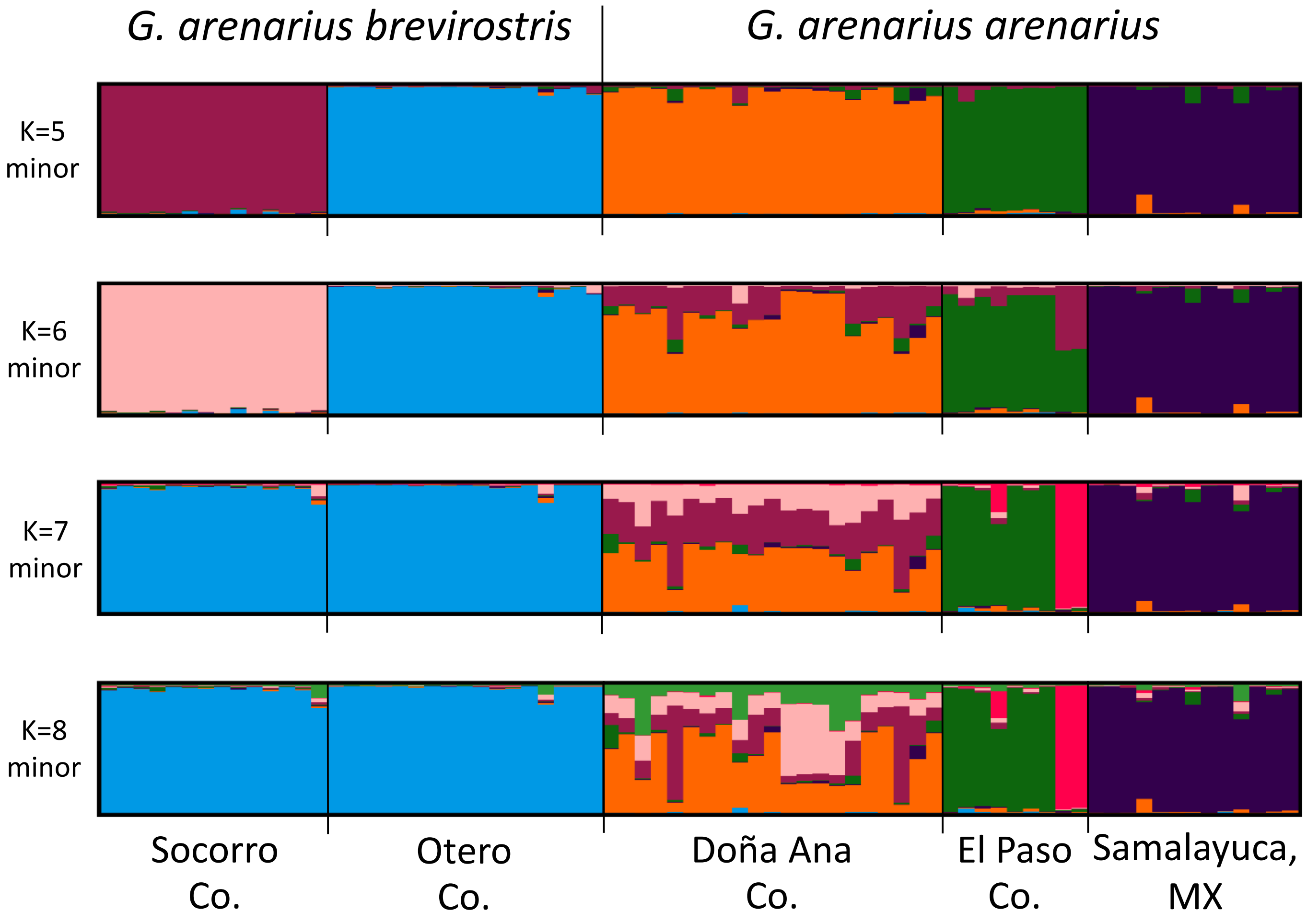

Supporting Information Figure 1. STRUCTURE minor modalities.

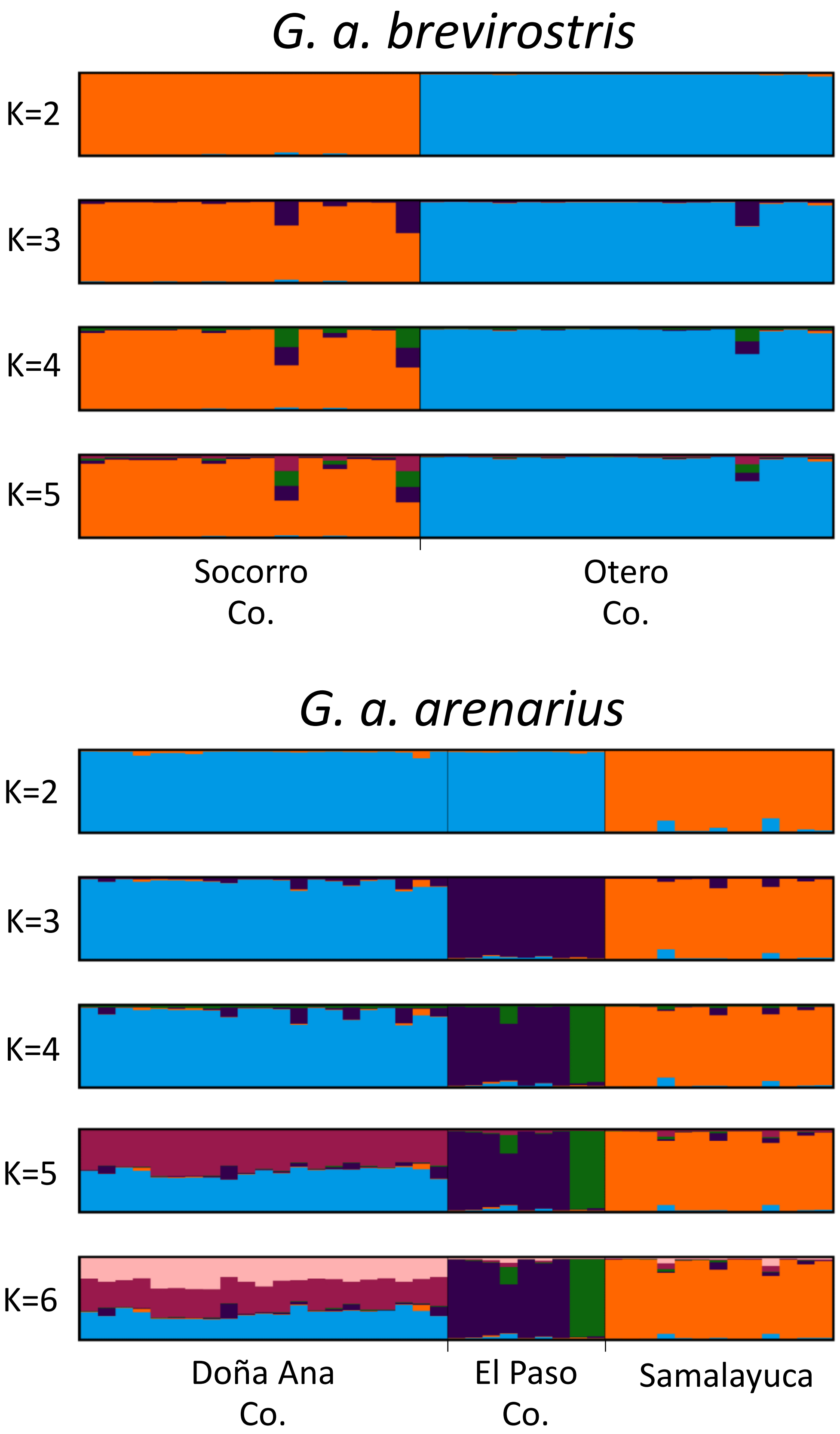

Supporting Information Figure 2. Hierarchical STRUCTURE analysis performed for each of the two groups defined by K=2 (the two groups corresponded to current subspecies designations).
