# Supplementary material for: Population genetic structure of a Chihuahuan Desert endemic mammal, the desert pocket gopher, Geomys arenarius
Source: Ecol Evol. 2023 Sep 28;13(10):e10576. doi: 10.1002/ece3.10576 (PMC10539045; doi:10.1002/ece3.10576)
Supplement: Supplementary file 2 — Appendix S1. [file ECE3-13-e10576-s003.docx]

Appendix 1. The 75 individuals included in this study are listed below and are housed at the Museum of Southwestern Biology, University of New Mexico (MSB) or the Colección Científica de Vertebrados, Universidad Autónoma de Ciudad Juárez (CCV UACJ). Map symbol numbers refer to those in Figure 1.

| Accession | Species/Subspecies | Genbank | Haplotype | County, State | Map symbol | Locality | Latitude | Longitude |
| --- | --- | --- | --- | --- | --- | --- | --- | --- |
| MSB 271403 | G. a. arenarius | MW558533 | 3 | Doña Ana, NM | 4 | E Bank of Rio Grande, W Las Cruces |  |  |
| MSB 271404 | G. a. arenarius | MW558534 | 3 | Doña Ana, NM | 4 | E Bank of Rio Grande, W Las Cruces |  |  |
| MSB 271426 | G. a. arenarius | MW558535 | 3 | Doña Ana, NM | 4 | E Bank of Rio Grande, W Las Cruces |  |  |
| MSB 271427 | G. a. arenarius | MW558536 | 3 | Doña Ana, NM | 4 | E Bank of Rio Grande W Las Cruces |  |  |
| MSB 271219 | G. a. arenarius | MW558542 | 3 | Doña Ana, NM | 4 | Las Cruces, E bank of Rio Grande |  |  |
| MSB 271220 | G. a. arenarius | MH319098 | 1 | Doña Ana, NM | 4 | Las Cruces, E bank of Rio Grande |  |  |
| MSB 271221 | G. a. arenarius | MH319062 | N/A | Doña Ana, NM | 4 | Las Cruces, E bank of Rio Grande |  |  |
| MSB 333041 | G. a. arenarius | MW558504 | 2 | Doña Ana, NM | 4 | Las Cruces, La Llorona Park, E. bank of Rio Grande River | 32.308715 | -106.825746 |
| MSB 333031 | G. a. arenarius | MW558505 | 3 | Doña Ana, NM | 4 | Las Cruces, La Llorona Park, E. bank of Rio Grande River | 32.308715 | -106.825746 |
| MSB 333033 | G. a. arenarius | MW558506 | 2 | Doña Ana, NM | 4 | Las Cruces, La Llorona Park, E. bank of Rio Grande River | 32.308715 | -106.825746 |
| MSB 333044 | G. a. arenarius | MW558507 | N/A | Doña Ana, NM | 4 | Las Cruces, La Llorona Park, E. bank of Rio Grande River | 32.308715 | -106.825746 |
| MSB 333035 | G. a. arenarius | MW558508 | 4 | Doña Ana, NM | 5 | 3 mi NE Anthony | 32.0257656 | -106.648886 |
| MSB 333047 | G. a. arenarius | MW558509 | 4 | Doña Ana, NM | 5 | 3 mi NE Anthony | 32.0257656 | -106.648886 |
| MSB 333042 | G. a. arenarius | MW558510 | 4 | Doña Ana, NM | 5 | 3 mi NE Anthony | 32.0257656 | -106.648886 |
| MSB 333043 | G. a. arenarius | MW558511 | 5 | Doña Ana, NM | 4 | 9 mi S, 1 mi E Las Cruces | 32.1818047 | -106.7461921 |
| MSB 270863 | G. a. arenarius | MH319100 | N/A | Doña Ana, NM | 4 | East Bank Rio Grande, Las Cruces |  |  |
| MSB 333026 | G. a. arenarius | MW558557 | 3 | Doña Ana, NM | 4 | Las Cruces, E. Bank Rio Grande | 32.30648432 | -106.8244726 |
| MSB 333027 | G. a. arenarius | MW558558 | 2 | Doña Ana, NM | 4 | Las Cruces, E. Bank Rio Grande | 32.30548622 | -106.8242614 |
| MSB 333020 | G. a. arenarius | MW558559 | 3 | Doña Ana, NM | 4 | Las Cruces, E. Bank Rio Grande | 32.30932341 | -106.825857 |
| MSB 333028 | G. a. arenarius | MW558560 | 2 | Doña Ana, NM | 4 | Las Cruces, E. Bank Rio Grande | 32.30518473 | -106.8239049 |
| MSB 333056 | G. a. arenarius | MW558561 | 2 | Doña Ana, NM | 4 | Las Cruces, E. Bank Rio Grande | 32.30077371 | -106.8282979 |
| MSB 271222 | G. a. arenarius | MW558543 | 13 | El Paso, TX | 7 | 4 mi. SE Fabens |  |  |
| MSB 270864 | G. a. arenarius | N/A | 15 | El Paso, TX | 7 | 4 mi SE Fabens |  |  |
| MSB 270865 | G. a. arenarius | MH319087 | N/A | El Paso, TX | 6 | 1 mi SE Fabens |  |  |
| MSB 333038 | G. a. arenarius | MW558551 | 13 | El Paso, TX | 7 | 2.7 mi SE Fabens |  |  |
| MSB 333022 | G. a. arenarius | MW558552 | 18 | El Paso, TX | 7 | 2.7 mi SE Fabens |  |  |
| MSB 333039 | G. a. arenarius | MW558553 | 19 | El Paso, TX | 6 | 1 mi SE Fabens |  |  |
| MSB 333025 | G. a. arenarius | MW558554 | 18 | El Paso, TX | 7 | 2.7 mi SE Fabens |  |  |
| MSB 333024 | G. a. arenarius | MW558555 | 15 | El Paso, TX | 7 | 2.7 mi SE Fabens |  |  |
| MSB 333023 | G. a. arenarius | MW558556 | 20 | El Paso, TX | 7 | 2.7 mi SE Fabens |  |  |
| MSB 333050 | G. a. brevirostris | MW558503 | N/A | Otero, NM | 3 | White Sands National Monument | 32.76744683 | -106.1868944 |
| MSB 333052 | G. a. brevirostris | MW558514 | 1 | Otero, NM | 3 | White Sands National Monument | 32.76744683 | -106.1868944 |
| MSB 333036 | G. a. brevirostris | N/A | N/A | Otero, NM | 3 | White Sands National Monument | 32.7907552 | -106.2244512 |
| MSB 333055 | G. a. brevirostris | N/A | N/A | Otero, NM | 3 | White Sands National Monument | 32.7907552 | -106.2244512 |
| MSB 333045 | G. a. brevirostris | N/A | N/A | Otero, NM | 3 | White Sands National Monument | 32.7907552 | -106.2244512 |
| MSB 333051 | G. a. brevirostris | MW558515 | 1 | Otero, NM | 3 | White Sands National Monument | 32.76744683 | -106.1868944 |
| MSB 333053 | G. a. brevirostris | MW558516 | 1 | Otero, NM | 3 | White Sands National Monument | 32.76744683 | -106.1868944 |
| MSB 333029 | G. a. brevirostris | MW558517 | 1 | Otero, NM | 3 | White Sands National Monument | 32.79046979 | -106.222995 |
| MSB 333034 | G. a. brevirostris | MW558518 | 1 | Otero, NM | 3 | White Sands National Monument | 32.79075321 | -106.2244512 |
| MSB 333054 | G. a. brevirostris | MW558519 | 7 | Otero, NM | 3 | White Sands National Monument | 32.78930301 | -106.2224024 |
| MSB 333040 | G. a. brevirostris | N/A | N/A | Otero, NM | 3 | White Sands National Monument | 32.79081094 | -106.2231599 |
| MSB 273683 | G. a. brevirostris | MH319097 | N/A | Otero, NM | 3 | 0.5 mi WNW Visitor Center, White Sands Natl. Mon. |  |  |
| MSB 271154 | G. a. brevirostris | MW558540 | 1 | Otero, NM | 3 | 18 mi. SW Alamogordo |  |  |
| MSB 271155 | G. a. brevirostris | MW558541 | N/A | Otero, NM | 3 | 18 mi. SW Alamogordo |  |  |
| MSB 270860 | G. a. brevirostris | MH319095 | 1 | Otero, NM | 3 | 18 mi SW Alamogordo, White Sands National Monument |  |  |
| MSB 270861 | G. a. brevirostris | MH319086 | 1 | Otero, NM | 3 | 18 mi SW Alamogordo, White Sands National Monument |  |  |
| MSB 270862 | G. a. brevirostris | N/A | 1 | Otero, NM | 3 | 18 mi SW Alamogordo, White Sands National Monument |  |  |
| MSB 333037 | G. a. brevirostris | MW558512 | 6 | Socorro, NM | 1 | 1.5 mi S Gran Quivira Unit of Salinas Pueblo Missions National Monument | 34.2385049 | -106.0983915 |
| MSB 333046 | G. a. brevirostris | MW558513 | 6 | Socorro, NM | 1 | 1.5 mi S Gran Quivira Unit of Salinas Pueblo Missions National Monument | 34.2385049 | -106.0983915 |
| MSB 271130 | G. a. brevirostris | MW558537 | 6 | Socorro, NM | 1 | 2 mi. S Gran Quivira |  |  |
| MSB 271131 | G. a. brevirostris | MH319044 | N/A | Socorro, NM | 1 | 2 mi. S Gran Quivira |  |  |
| MSB 271132 | G. a. brevirostris | MW558538 | 6 | Socorro, NM | 1 | 2 mi. S Gran Quivira |  |  |
| MSB 271133 | G. a. brevirostris | MW558539 | 6 | Socorro, NM | 1 | 2 mi. S Gran Quivira |  |  |
| MSB 270836 | G. a. brevirostris | MH319032 | 14 | Socorro, NM | 1 | 2 mi S Gran Quivira Unit of Salinas Pueblo Missions National Monument |  |  |
| MSB 270837 | G. a. brevirostris | MH319048 | 6 | Socorro, NM | 1 | 2 mi S Gran Quivira Unit of Salinas Pueblo Missions National Monument |  |  |
| MSB 270893 | G. a. brevirostris | MH319033 | 17 | Socorro, NM | 2 | 1.5 mi S, 11.5 mi E San Antonio |  |  |
| MSB 333021 | G. a. brevirostris | MW558562 | 21 | Socorro, NM | 1 | 2 mi S Gran Quivira Unit of Salinas Pueblo Missions National Monument | 34.23275057 | -106.0993483 |
| MSB 333048 | G. a. brevirostris | MW558563 | 14 | Socorro, NM | 1 | 2 mi S Gran Quivira Unit of Salinas Pueblo Missions National Monument | 34.23093037 | -106.1001932 |
| MSB 333011 | G. a. brevirostris | MW558564 | 22 | Socorro, NM | 1 | 2 mi S Gran Quivira Unit of Salinas Pueblo Missions National Monument | 34.23346641 | -106.0989667 |
| MSB 333049 | G. a. brevirostris | MW558565 | 21 | Socorro, NM | 1 | 2 mi S Gran Quivira Unit of Salinas Pueblo Missions National Monument | 34.23078727 | -106.1000611 |
| MSB 333032 | G. a. brevirostris | MW558566 | 14 | Socorro, NM | 1 | 2 mi S Gran Quivira Unit of Salinas Pueblo Missions National Monument | 34.2341842 | -106.098368 |
| CCV UACJ 663 | G. a. arenarius | MW558520 | 8 | El Municipio de Juarez | 8 | 10 mi S Samalayuca | 31.20314 | -106.5 |
| CCV UACJ 664 | G. a. arenarius | MW558521 | 9 | El Municipio de Juarez | 8 | 10 mi S Samalayuca | 31.202 | -106.4926 |
| CCV UACJ 665 | G. a. arenarius | MW558522 | 10 | El Municipio de Juarez | 8 | 10 mi S Samalayuca | 31.20131 | -106.4926 |
| CCV UACJ 666 | G. a. arenarius | MW558523 | 11 | El Municipio de Juarez | 8 | 10 mi S Samalayuca | 31.2 | -106.4925 |
| CCV UACJ 667 | G. a. arenarius | MW558524 | 12 | El Municipio de Juarez | 8 | 10 mi S Samalayuca | 31.2 | -106.49 |
| CCV UACJ 668 | G. a. arenarius | MW558525 | 11 | El Municipio de Juarez | 8 | 10 mi S Samalayuca | 31.2 | -106.49 |
| CCV UACJ 669 | G. a. arenarius | MW558526 | 10 | El Municipio de Juarez | 8 | 10 mi S Samalayuca | 31.2 | -106.49 |
| CCV UACJ 670 | G. a. arenarius | MW558527 | 12 | El Municipio de Juarez | 8 | 10 mi S Samalayuca | 31.20242 | -106.4926 |
| CCV UACJ 671 | G. a. arenarius | MW558528 | 8 | El Municipio de Juarez | 8 | 10 mi S Samalayuca | 31.20228 | -106.4927 |
| CCV UACJ 672 | G. a. arenarius | MW558529 | 10 | El Municipio de Juarez | 8 | 10 mi S Samalayuca | 31.20017 | -106.4925 |
| CCV UACJ 673 | G. a. arenarius | MW558530 | 8 | El Municipio de Juarez | 8 | 10 mi S Samalayuca | 31.2015 | -106.4926 |
| CCV UACJ 674 | G. a. arenarius | MW558531 | 8 | El Municipio de Juarez | 8 | 10 mi S Samalayuca | 31.19978 | -106.4925 |
| CCV UACJ 675 | G. a. arenarius | MW558532 | 12 | El Municipio de Juarez | 8 | 10 mi S Samalayuca | 31.19953 | -106.4925 |
| MSB 270888 | G. knoxjonesi | MW558567 | N/A | Eddy | N/A | 1 mi. N, 9.5 mi. E Loco Hills |  |  |
