# Supplementary material for: Population genetic structure of a Chihuahuan Desert endemic mammal, the desert pocket gopher, Geomys arenarius
Source: Ecol Evol. 2023 Sep 28;13(10):e10576. doi: 10.1002/ece3.10576 (PMC10539045; doi:10.1002/ece3.10576)
Supplement: Supplementary file 3 — Table S1. [file ECE3-13-e10576-s002.docx]

Supporting Information

Population genetic structure of a ChihuahuanDesert endemic, the desert pocket gopher, *Geomysarenarius.*

Ecology and Evolution

Russell S. Pfau1*, Ashley N. Kozora2, Ana B. Gatica-Colima3, Philip S. Sudman1

1Department of Biological Sciences, Tarleton State University, Stephenville, Texas 76402, U.S.A., pfau@tarleton.edu

2Cooper High School, Abilene, Texas 79605, U.S.A., ashley.kozora@abileneisd.org

3Departamentode CienciasQuímico-Biológicas, Instituto de CienciasBiomédicas, Universidad Autónomade Ciudad Juárez, Anillo Envolventedel PRONAF y Estocolmos/n, Ciudad Juárez, Chih. 32310, Mexico, agatica@uacj.mx

Supporting Information Table 1. Occurrence data of museum specimens with precise locality data used to document soil mapping units inhabited by *G. arenarius*. Specimens are housed at the Museum of Southwestern Biology, University of New Mexico (MSB) or University of Texas—El Paso (UTEP).

| MuseumAccession | Species/Subspecies | County, State | Locality | Latitude | Longitude | Soil mapping units |
| --- | --- | --- | --- | --- | --- | --- |
| MSB 333037, 333046 | *G. a. brevirostris* | Socorro, NM | 1.5 mi S Gran Quivira Unit of Salinas Pueblo Missions National Monument | 34.2385049 | -106.0983915 | 818-Mespun fine sand |
| MSB 333021 | *G. a. brevirostris* | Socorro, NM | 2 mi S Gran Quivira Unit of Salinas Pueblo Missions National Monument | 34.23275057 | -106.0993483 | 818-Mespun fine sand |
| MSB 333048 | *G. a. brevirostris* | Socorro, NM | 2 mi S Gran Quivira Unit of Salinas Pueblo Missions National Monument | 34.23093037 | -106.1001932 | 818-Mespun fine sand |
| MSB 333011 | *G. a. brevirostris* | Socorro, NM | 2 mi S Gran Quivira Unit of Salinas Pueblo Missions National Monument | 34.23346641 | -106.0989667 | 818-Mespun fine sand |
| MSB 333049 | *G. a. brevirostris* | Socorro, NM | 2 mi S Gran Quivira Unit of Salinas Pueblo Missions National Monument | 34.23078727 | -106.1000611 | 818-Mespun fine sand |
| MSB 333032 | *G. a. brevirostris* | Socorro, NM | 2 mi S Gran Quivira Unit of Salinas Pueblo Missions National Monument | 34.2341842 | -106.098368 | 818-Mespun fine sand |
| MSB 333050, 333051, 333052, 333053 | *G. a. brevirostris* | Otero, NM | White Sands National Monument | 32.76744683 | -106.1868944 | 43-Lark-Transformer association (gypsiferous sand) |
| MSB 333036, 333045, 333055 | *G. a. brevirostris* | Otero, NM | White Sands National Monument | 32.7907552 | -106.2244512 | 9-Astrobee-Lark association (gypsiferous sand) |
| MSB 333029 | *G. a. brevirostris* | Otero, NM | White Sands National Monument | 32.79046979 | -106.222995 | 9-Astrobee-Lark association (gypsiferous sand) |
| MSB 333034 | *G. a. brevirostris* | Otero, NM | White Sands National Monument | 32.79075321 | -106.2244512 | 9-Astrobee-Lark association (gypsiferous sand) |
| MSB 333054 | *G. a. brevirostris* | Otero, NM | White Sands National Monument | 32.78930301 | -106.2224024 | 9-Astrobee-Lark association (gypsiferous sand) |
| MSB 333040 | *G. a. brevirostris* | Otero, NM | White Sands National Monument | 32.79081094 | -106.2231599 | 9-Astrobee-Lark association (gypsiferous sand) |
| MSB 333031, 333033, 333041, 333044 | *G. a. arenarius* | Doña Ana, NM | Las Cruces, La Llorona Park, E. bank of Rio Grande River | 32.308715 | -106.825746 | Br-Brazito loamy fine sand |
| MSB 333035, 333042, 333047 | *G. a. arenarius* | Doña Ana, NM | 3 mi NE Anthony | 32.0257656 | -106.648886 | On boundary between Ap-Anthony-Vinton fine sandy loams, Ag-Agua loam, and Hg-Harkey loam |
| MSB 333043 | *G. a. arenarius* | Doña Ana, NM | 9 mi S, 1 mi E Las Cruces | 32.1818047 | -106.7461921 | Ge-Glendale loam  Adjacent to Ag-Agua loam and Ar-Anthony-Vinton loams |
| MSB 333026 | *G. a. arenarius* | Doña Ana, NM | Las Cruces, E. Bank Rio Grande | 32.30648432 | -106.8244726 | Ah-Agua clay loam  Adjacent to Bs-Brazito very fine sand loam |
| MSB 333027 | *G. a. arenarius* | Doña Ana, NM | Las Cruces, E. Bank Rio Grande | 32.30548622 | -106.8242614 | Bs-Brazito very fine sand loam |
| MSB 333020 | *G. a. arenarius* | Doña Ana, NM | Las Cruces, E. Bank Rio Grande | 32.30932341 | -106.825857 | Br-Brazito loamy fine sand |
| MSB 333028 | *G. a. arenarius* | Doña Ana, NM | Las Cruces, E. Bank Rio Grande | 32.30518473 | -106.8239049 | Bs-Brazito loamy fine sand |
| UTEP 1895 | *G. a. arenarius* | Doña Ana, NM | Ditch at end of Frontera Rd., NW El Paso | 31.8071409 | -106.5823408 | Ap-Anthony-Vinton fine sandy loams |
| UTEP 5701 | *G. a. arenarius* | El Paso, TX | Border hwy behind Ascarate golf course, El Paso | 31.750254 | -106.411057 | Mg-Made land, Gila soil material |
| UTEP 7423 | *G. a. arenarius* | El Paso, TX | Socorro, 665 Stedham Circle (residence) | 31.670723 | -106.280154 | Sc-Saneli silty clay  Adjacent to Vn-Vinton fine sandy loam and Br-Brazito loamy fine sand |
| UTEP 7545 | *G. a. arenarius* | El Paso, TX | Along levee of Rio Grande, 100 m upstream from Country Club Rd | 31.8475348 | -106.6066209 | Mg-Made land, Gila soil material  Adjacent to Ha-Harkey loam and Bs-Brazito loamy fine sand |
| UTEP 1397 | *G. a. arenarius* | El Paso, TX | Residence, 428 Lindbergh Ave., El Paso, upper valley | 31.8484325 | -106.5931343 | Ha-Harkey loam |
| UTEP 22 | *G. a. arenarius* | El Paso, TX | Residence, El Paso, 803 Mamie Rd, N of El Paso, Rio Grande valley | 31.8593111 | -106.6015151 | Ga-Gila fine sandy loam |
